# Fabrication of a Human Skin Mockup with a Multilayered Concentration Map of Pigment Components Using a UV Printer

**DOI:** 10.3390/jimaging8030073

**Published:** 2022-03-15

**Authors:** Kazuki Nagasawa, Shoji Yamamoto, Wataru Arai, Kunio Hakkaku, Chawan Koopipat, Keita Hirai, Norimichi Tsumura

**Affiliations:** 1 Graduate School of Science and Engineering, Chiba University, Chiba 263-8522, Japan; hirai@faculty.chiba-u.jp (K.H.); tsumura@faculty.chiba-u.jp (N.T.); 2 Tokyo Metropolitan College of Industrial Technology, Tokyo 140-0011, Japan; yamasho@g.metro-cit.ac.jp; 3 MIMAKI ENGINEERING Co., Ltd., Nagano 389-0512, Japan; wataru_arai@mimaki.com (W.A.); kunio_hakkaku@mimaki.com (K.H.); 4 Department of Photographic Science and Printing Technology, Chulalongkorn University, Bangkok 10330, Thailand; chawan.k@chula.ac.th

**Keywords:** 3D printing, human skin, skin pigments, image processing, machine learning

## Abstract

In this paper, we propose a pipeline that reproduces human skin mockups using a UV printer by obtaining the spatial concentration map of pigments from an RGB image of human skin. The pigment concentration distributions were obtained by a separating method of skin pigment components with independent component analysis from the skin image. This method can extract the concentration of melanin and hemoglobin components, which are the main pigments that make up skin tone. Based on this concentration, we developed a procedure to reproduce a skin mockup with a multi-layered structure that is determined by mapping the absorbance of melanin and hemoglobin to CMYK (Cyan, Magenta, Yellow, Black) subtractive color mixing. In our proposed method, the multi-layered structure with different pigments in each layer contributes greatly to the accurate reproduction of skin tones. We use a UV printer because the printer is capable of layered fabrication by using UV-curable inks. As the result, subjective evaluation showed that the artificial skin reproduced by our method has a more skin-like appearance than that produced using conventional printing.

## 1. Introduction

In recent years, the usage of 3D printers has increased in a wide range of fields, such as medicine and dentistry [1,2], for prototyping of automobile parts [3], and for manufacturing of consumer products; they are also now used in general households. In particular, modeling of character figures for general consumers and the reproduction of historical artworks are highly demanded applications in 3D printing. These applications require accurate reproduction of not only shape but also appearance, including color, surface and material properties. Accurate reproduction of appearance in 3D printing is difficult in conventional 3D printing machines that use a limited number of materials; however, since the development of multi-material 3D printers, various studies were conducted on appearance reproduction recently [4].

Several studies on appearance reproduction in 3D printing are described below. In color reproduction, some studies applied error-diffusion halftoning—a technique that enables smooth tonal representation in 2D printing and 3D printing [5,6]. This method enables a detailed representation of tone in 3D printing, which is limited to 3D materials and inks. However, the method using halftone dots causes artifacts that arise out of the manifestation of each ink dot, so there is research on color 3D printing using contoning, a method of layering various inks [7]. Furthermore, a study reproduced not only color reproduction but also spectral reflectance by using multilayer inks that combine halftoning and contoning [8]. There are studies that reproduced gloss as a material appearance [9,10]. These methods involve adjusting the printing parameters of the 3D printer or applying varnish in high resolution on the surface. In addition, many studies have focused on material appearance, particularly the control of translucency. For example, one study proposed a method to reproduce complex scattering properties [11], whereby the scattering properties of several materials are measured and radial reflection and scattering profiles are generated. This allows for the proper arrangement of the material in the depth direction and reproduces complex scattering properties well. In another study, complex light scattering was reproduced using a bidirectional scattering-surface reflectance distribution function (BSSRDF), which is a function that represents surface-subsurface scattering [12]. In this study, materials with different scattering effects were stacked with varying spatial thicknesses to represent inhomogeneous scattering. However, in modeling with translucent materials, there is a problem of the loss of detailed surface texture information due to subsurface scattering. This problem was overcome using an inverse Monte Carlo simulation-based method to optimize the material arrangement under the surface problem [13]. An alternative approach to fabricate translucent objects is to use mixtures of translucent materials [14]. In this approach, the concentration of the mixture of several translucent materials is estimated such that it reproduces the desired appearance and scattering properties. Furthermore, a method to perform full-color modeling with spatially varying translucency was recently proposed [15] using RGBA (Red, Green, Blue, Alpha) signals instead of BSSRDF, which has high measurement and processing costs (the “A” in RGBA is the signal for translucency). The accuracy of this method was subsequently enhanced [16] by optimizing the signal to link to both optical material properties and human perceptual uniformity, independent of hardware and software. On the other hand, another study has reproduced arbitrary modulation transfer functions on the object by combining translucent materials such as erasers or wax with UV ink [17].

As described above, various studies were performed to reproduce translucent appearance. In this paper, we focus on a typical translucent material, human skin. Reproduction of realistic skin appearance provides value-added products for character figures and so on. In the field of computer graphics, the reproduction of human skin is also important, and many methods and techniques have been developed, and the representative studies use multi-layered translucent materials in the simulation [18,19]. Moreover, skin appearance is also important in the medical and cosmetic fields. In these fields, it is known that human skin has a layered structure consisting of an epidermal layer containing melanin pigments and a dermal layer containing hemoglobin pigments [20]. Since these pigments are the elements that determine skin color, blood volume and oxygen saturation can be estimated from skin color [21,22]. Furthermore, the relationship between pigment and appearance is also used in the development of cosmetics [23]. In 3D printing, the reproduction of skin appearance with pigment information as a real object is very useful in the fields described above. As already mentioned above, skin is a multilayered structure with different pigments in each layer, and there are studies that have attempted to reproduce this structure in 3D printing. One study used multilayered skin modeling performed with a neural network-based method to estimate the optimal layer structure layout for reproducing human skin with arbitrary skin color and transparency [24]. Furthermore, a study has succeeded in reducing the fabrication cost by using simulation data [25]. However, these studies assumed that the skin is a uniform surface, whereas in reality, its color varies spatially. To solve this problem, it is necessary to reproduce the spatial variation of skin color.

In the present paper, we propose a pipeline for 3D modeling of human skin with multilayered spatial distribution of pigments, using skin images taken by an RGB (red, green, blue) camera. The coloring layer consisted of two layers—the epidermal layer with melanin pigments and the dermal layer with hemoglobin pigment—using a technique called pigment component separation [26]. Conventionally, light scattering simulation methods such as the Monte Carlo method are used to simulate the color of objects with a multi-layered structure [27]. The optimization is expected to be performed by a non-linear optimization technique to reproduce appropriate skin tone; however, it requires huge calculations to obtain the spatial distribution of color on the skin. Therefore, we used a simple light scattering simulation method based on the modified Lambert–Beer law, and extracted the spatial distribution of hemoglobin and melanin from the skin color image [26]. The extracted spatial distribution of hemoglobin and melanin were converted to CMYK values by a color patch-based method and used in the 3D printer while empirically inserting the clear layer to reproduce the skin appropriately. The clear layer was made with a clear ink that is a varnish-like ink that does not contain pigments. We used Mimaki’s clear ink (SPC-0659CL, Mimaki Engineering, Nagano, Japan) in this study. In contrast to most research in this area, which is limited to synthetic inputs, we propose an end-to-end reproduction method for real skin. It is also novel in that it uses pigment concentration to represent skin tone.

In Section 4, we describe the creation of a color patch to convert pigment concentrations to CMYK values. In Section 5, we describe the conversion method. In Section 6, we describe the process of modeling human skin using a 3D printer, and in Section 7, we present the results of the subjective evaluation experiment.

## 2. Previous Works

### 2.1. Skin Pigment Separation

The color of human skin is determined by two main pigments, melanin and hemoglobin [28,29]. The concentration of each pigment varies between individuals as well as between parts of the body. Although there are various techniques to measure the concentration of pigment components in human skin, Tsumura et al. proposed an efficient method using common RGB images [16]. They used independent component analysis to separate the RGB signal into melanin and hemoglobin components. This skin model assumes that the boundaries of each layer of the skin are flat, and that melanin is exclusively present in the epidermis and hemoglobin is exclusively present in the dermis. Considering the light incident on this skin model, it can be divided into surface reflected light, which is reflected on the surface of the skin, and internally reflected light, which is repeatedly absorbed, scattered, and emitted inside the skin. Only internally reflected light is used as an observation signal because the light scattered and absorbed by skin pigment expresses the skin color [28,29].

To separate the RGB signal into melanin and hemoglobin signals, it is necessary to estimate the melanin and hemoglobin vectors that constitute the plane of skin color distribution. First, the skin color distribution plane consisting of the first and second principal component vectors is estimated by principal component analysis of the skin image in a small region that is less affected by changes in illumination intensity. This is based on the assumption that the observed signal in this region resides on the skin color distribution plane. Next, the melanin and hemoglobin vectors are estimated using independent component analysis. Finally, the pigment concentration is obtained by projecting the observed signal to each pigment vector.

**Figure 1:**
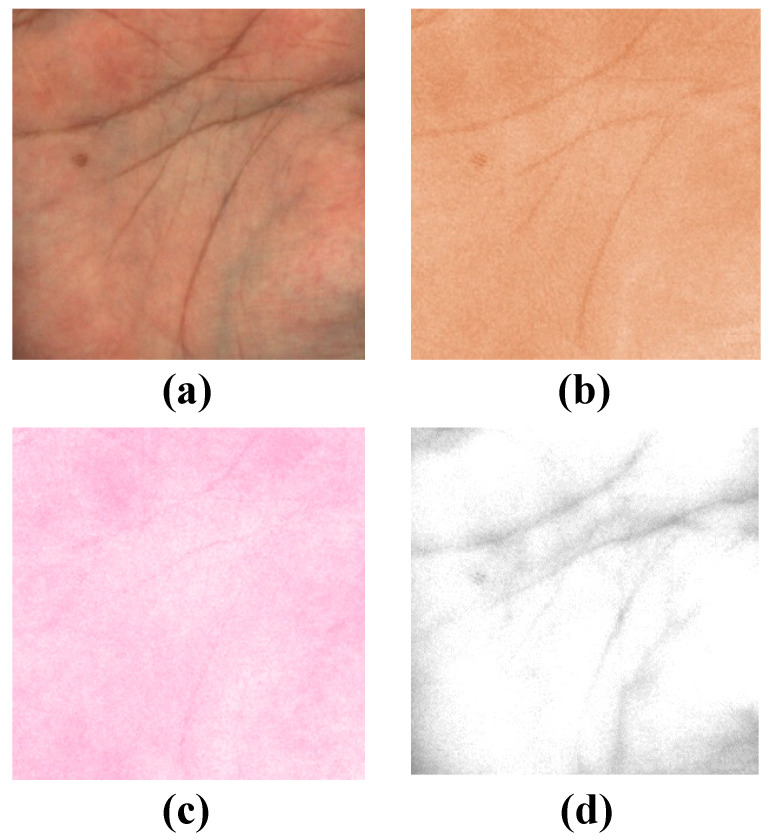
Results of pigment component separation for a skin image: (**a**) original image; (**b**) melanin component; (**c**) hemoglobin component; (**d**) shading.

Tsumura et al. also proposed a method to eliminate the effects of shading due to the shape of the skin. Assuming that the skin color distribution plane is obtained by the previously described method, they used the fact that the intensity of the shading changes in the same direction as the intensity of the illumination. First, the normal vector of the skin color distribution plane was obtained and the distance from the observed signal was calculated. Then, the shading was removed by projecting the observed signal along the illumination intensity vector onto the skin color distribution plane. Figure 1 shows the results of applying the pigment component separation to the actual skin image. The melanin and hemoglobin components were extracted, and the shaded areas caused by unevenness were removed as shaded images.

### 2.2. Estimation of the Layered Ink Layout

**Figure 2:**
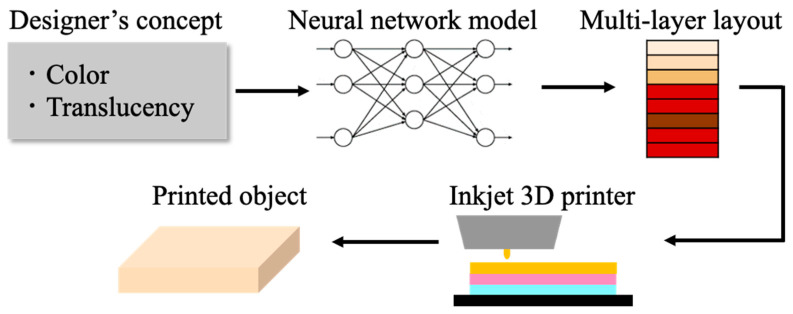
Overview of the method of Nagasawa et al. [24].

We referred to the method of Nagasawa et al., which predicts an appropriate multilayer ink layout for reproducing the texture of human skin by layering translucent ink [24]. They used the line spread function (LSF) measured from the skin created as a measure of translucency and used a neural network to estimate the layout. This pipeline enables designers to reproduce human skin with arbitrary skin color and transparency using a 3D printer. An overview of this method is shown in Figure 2. This method is based on an earlier study of painting reproduction using multispectral data [8], whereby the neural network learned by obtaining spectral reflectance from 20,878 multilayer patches. Nagasawa et al. followed this method to create color patches that approximate the human skin. Their structure consisted of three different layers, which were created by changing the conditions, such as the order of the clear layer, skin color layer, and red layer from the top.

In this method, the information about translucency and skin color is expressed using the LSF. Therefore, it is necessary to measure the LSF from the created color patch. Nagasawa et al. first irradiated the patch with an edge image that illuminated only half of the patch before taking the image. They calculated the LSF by obtaining the transitions of pixel values in the captured image. The difference between the calculated LSF and the layout of each patch was then used to train an encoder–decoder type neural network [30], in which the LSF was both the input and output, and the layout was obtained as an intermediate output. Finally, a pipeline that outputs the optimal layout for 3D printing was obtained using the LSF measured from the CG of human skin with arbitrary skin color and translucency. However, this method does not take into account the spatial distribution of the pigments. Therefore, in our study, we tried to reproduce human skin with multilayered spatial distribution of pigments.

## 3. Overview of the Proposed Method

**Figure 3:**
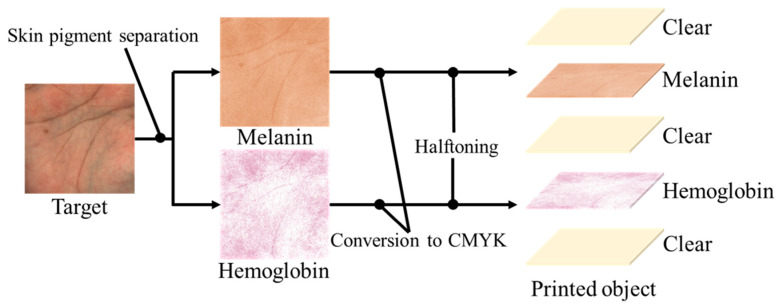
Our workflow for human skin reproduction with multilayer spatial distribution of pigment components.

In this paper, we introduce a workflow for creating realistic human skin with a 3D printer. This involves reproducing the spatial concentration distribution of pigments obtained from an image of human skin taken by an RGB camera. It is biologically known that skin has a layered structure, with an epidermal layer containing melanin pigments and a dermal layer containing hemoglobin pigments, and this model has been used in the fields of measurement and simulation [31]. Therefore, we consider that it is possible to reproduce appearance similar to actual skin by representing each pigment with ink and arranging them in a layered structure. The method is shown in Figure 3. The spatial concentration distributions of melanin and hemoglobin pigments were obtained by applying a pigment component separation method to the target skin image taken by an RGB camera. The method is robust to changes in illumination intensity because shading is removed at this time. For printing on an inkjet 3D printer, each pigment concentration was converted to a CMYK value. We obtained the transformation equation using a method with color patches. We used three methods: lookup tables, multiple regression analysis and neural networks. Then, a halftone process was applied to create a skin-like object consisting of a melanin layer as the epidermis and a hemoglobin layer as the dermis. Clear ink was inserted above and below these colored layers to improve the appearance. Furthermore, the thickness of this clear ink was adjusted and the thickness most suitable for the skin texture was determined by subjective evaluation.

## 4. Color Patches for Human Skin

### 4.1. Condition of Color Patches

**Figure 4:**
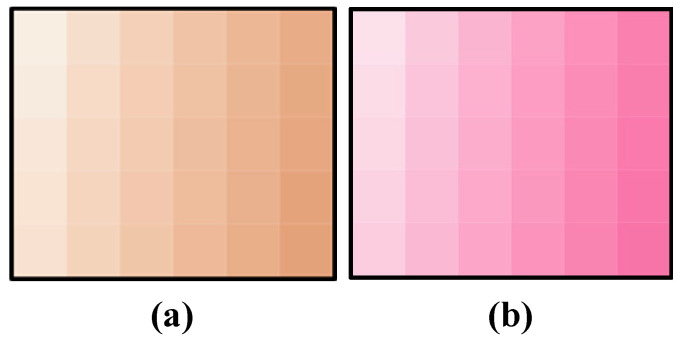
Pigment colors at 30 concentrations: (**a**) melanin component; (**b**) hemoglobin component.

To create a human skin color patch with a melanin and hemoglobin layer, we first determined the specification empirically through preliminary experiments as follows. This specification relates to the pigment concentrations and how they are combined. The colors of melanin and hemoglobin were obtained from their vectors, which were estimated by pigment component separation. As shown in Figure 4, 30 different concentrations of each pigment were extracted. The 30 concentrations were empirically determined to be in the range of Asian skin tones through printing experiments. By combining these concentrations, it is possible to create patches with 900 different concentration combinations. Since melanin and hemoglobin are the main factors that determine skin color, the color gamut of skin color can be covered by varying the concentration of the pigment color obtained by the method described in the reference [26]. The factor other than melanin that darkens the skin tone is shading, and since this study only targeted flat surfaces, we ignored these effects. The melanin and hemoglobin layers were used as the colored layers, and a white layer was placed at the bottom as a reflective layer. Here, white ink is the equivalent of a paper in 2D printing; it is very scattering and is used as a reflective layer. In addition, a clear layer was placed at three locations: at the top, between the hemoglobin and melanin layers, and between the hemoglobin and reflective layers.

### 4.2. Modeling of Color Patches

**Figure 5:**
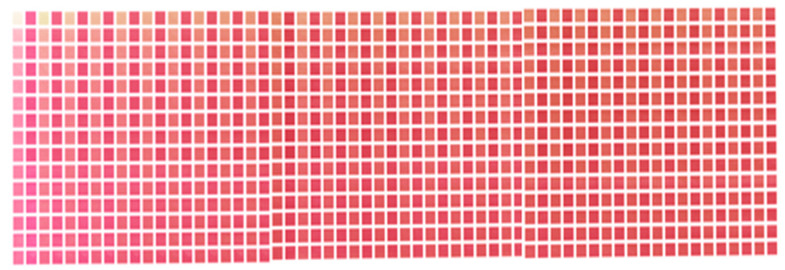
Color patches with two pigment layers and clear layers.

Because the color patches were made using an inkjet 3D printer, CMYK values were required to fabricate the color layer. The 30 pigment concentrations can be expressed as RGB values. Therefore, we transformed the color space with the ICC profile (Japan Color 2011, coated). In addition, halftoning was performed for the inkjet printing. The largest dot size was used to prevent unevenness in the stacking process. An error diffusion method (Floyd–Steinberg) was used for dithering to reduce the quantization error due to halftoning. The coefficients of error diffusion were 7/16, 3/16, 5/16 and 1/16 for the right neighbor, left bottom, true bottom and right bottom, respectively. Through these processes, 900 color patches with two pigment layers, as shown in Figure 5, were created. It can be seen that the skin tone changes significantly according to the change in pigment concentration. These patches cover a wide range of skin tones, from hypopigmented to hyperpigmented skin tones.

## 5. Conversion of Pigment Concentration to CMYK

**Figure 6:**
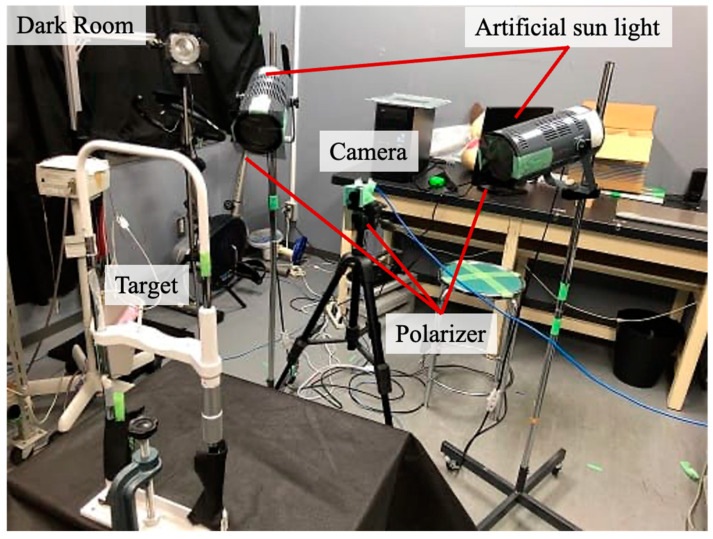
Experimental setup for capturing color patches.

The color patches were used to correlate pigment concentrations with CMYK values. Melanin and hemoglobin concentrations were obtained by applying pigment component separation to images taken of the 900 color patches. Figure 6 shows the experimental setup for capturing color patches. We used artificial sunlight as illumination in a dark room environment. A polarizer was placed in front of the camera and lighting when taking photos in order to remove surface reflections because only the internally scattered light is affected by pigments. We constructed a transformation method using the pigment concentration of each patch and the CMYK values calculated as described above. We used a lookup table (LUT), multiple regression analysis, and neural networks, and compared the accuracy of the printed results for these methods.

### 5.1. Lookup Table

The lookup table (LUT) is the simplest method that we used. The method stores 900 data points obtained from color patches and searches for the desired one. If there is no data point in the LUT that matches the target data point, the closest one is chosen by RMSE. The accuracy of the CMYK value prediction was verified by the cross-validation method. We used leave-one-out cross-validation, in which one data point was extracted from the dataset, and regression equations were trained with the other data and then verified with the extracted data [32]. The error was evaluated as the root mean square error (RMSE), which was 0.033.

### 5.2. Multiple Regression Analysis

Multiple regression analysis is a method of predicting a single objective variable by multiple explanatory variables. The objective variables are the CMYK values of the melanin and hemoglobin layers, which are designated as $D_{i,\;\mathrm{mel}},\;D_{i,\mathrm{hem}}\;\left(i=C,M,Y,K\right)$, respectively. The explanatory variables are $R_{\mathrm{mel}},G_{\mathrm{mel}},B_{\mathrm{mel}},R_{\mathrm{hem}},G_{\mathrm{hem}},B_{\mathrm{hem}}$, using the RGB value of the pigment concentration obtained by the separation of the pigment components. We used the RGB values for pigment concentration to provide redundancy and improve estimation accuracy. The regression equation by multiple regression analysis is as follows:

(1) $$\begin{array}{c} D_{i,\mathrm{mel}}=a_{R}\log R_{\mathrm{mel}}+a_{G}\log G_{\mathrm{mel}}+a_{B}\log B_{\mathrm{mel}}+b\\ i=C,M,Y,K \end{array}$$

(2) $$\begin{array}{c} D_{i,\mathrm{hem}}=a_{R}\log R_{\mathrm{hem}}+a_{G}\log G_{\mathrm{hem}}+a_{B}\log B_{\mathrm{hem}}+b\\ i=C,M,Y,K \end{array}$$

where $a_{R},a_{G},a_{B},b$ are the partial regression coefficients, which are optimized by learning. The accuracy of this method was verified by leave-one-out cross-validation, and the RMSE was 0.031 or 3.1%.

### 5.3. Neural Network

**Figure 7:**
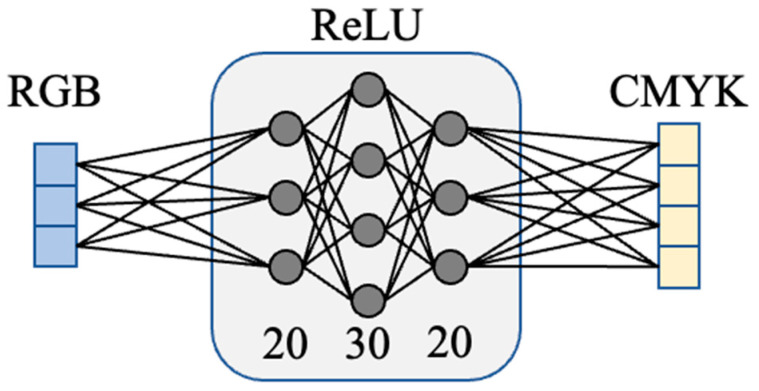
Illustration of our network structure.

**Figure 8:**
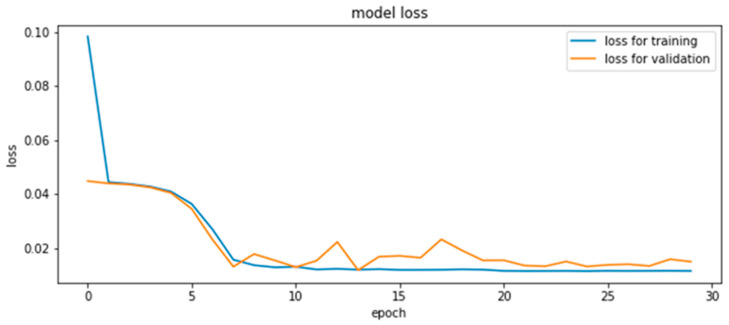
Transition of the training error and validation error in RMSE.

Here, we present the results of the estimation of CMYK values using the neural network. The structure of the model is shown in Figure 7. Two models with the same structure in each of the melanin and hemoglobin layers were constructed. The inputs were RGB values derived from the pigment concentration, and the outputs were CMYK values for printing. This was obtained by capturing 900 color patches and extracting the pigment components, and all 900 data points were used for training. The middle layer was the fully connected layer, which had 20, 30, and 20 neurons from the input side to the output side. ReLU (rectified linear unit) was used for the activation function and Adam was used for the optimization algorithm. As shown in the reference [33], it is common in the field of neural networks to use ReLU, and therefore we used ReLU empirically. We think it is required to compare with other activation functions in the future. The learning and validation errors for the 30-epoch learning are shown in Figure 8. The accuracy of this method was verified by leave-one-out cross-validation, and the RMSE was 0.035. The validation error during training was small around epoch 13; however, the error due to leave-one-out cross-validation was smallest at epoch 30.

## 6. Fabrication of Human Skin

### 6.1. Comparison of Methods

We will now describe the process of molding human skin with a multilayered spatial distribution of pigments using the three types of CMYK transformation method described in the previous section. First, we took images of the human target’s skin. The images were taken in a dark room with artificial sunlight. In the experiment, an industrial RGB camera (DKK33UP1300; The Imaging Source, New Taipei City, Taiwan) was used, and a polarizer was placed in front of the camera and lights to remove surface reflections. In addition, an image of a standard diffuse reflector was taken under the same conditions, while dividing the pixel values of the skin image by the pixel values of the diffuse reflector. This operation removes lighting effects approximately. Then, the melanin and hemoglobin concentrations of each pixel were obtained by the application of pigment component separation. Finally, the three different methods were used to estimate the CMYK values and halftoning was applied to the modeling. We targeted the palm of the hand, because the palm has a large spatial variation in pigment concentration, which allows us to better observe the spatial distribution of the pigment.

**Figure 9:**
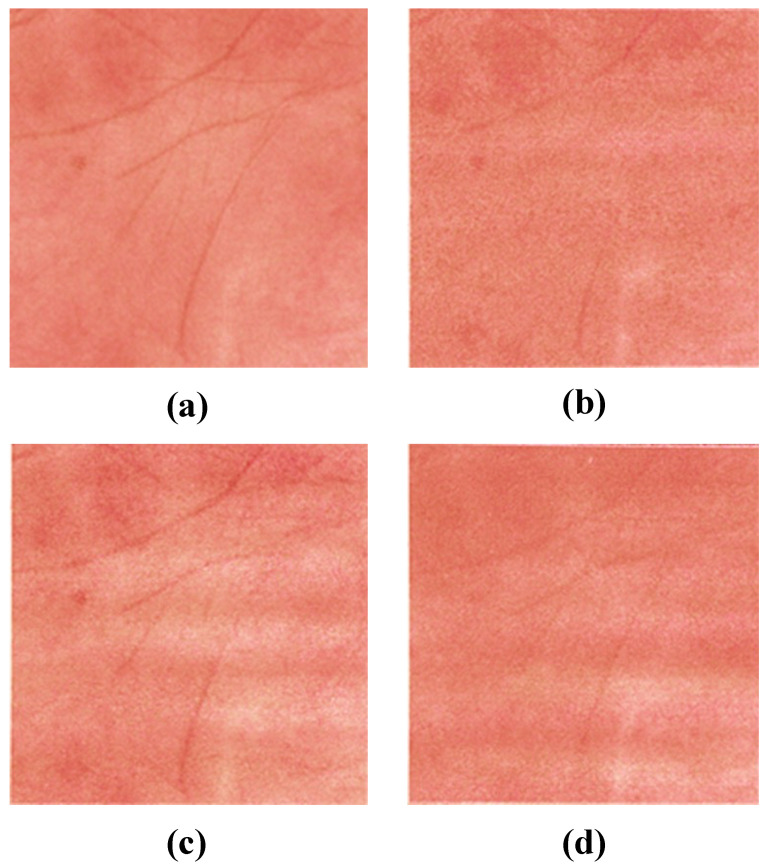
Results of fabrication using the three methods: (**a**) the target image; (**b**) result using the LUT; (**c**) result using multiple regression analysis; (**d**) result using the neural network. Compared to the original appearance, it can be seen that the fidelity of each method is different. Qualitatively, it is observed that the sample achieved by multiple regression analysis has a high reproducibility.

**Figure 10:**
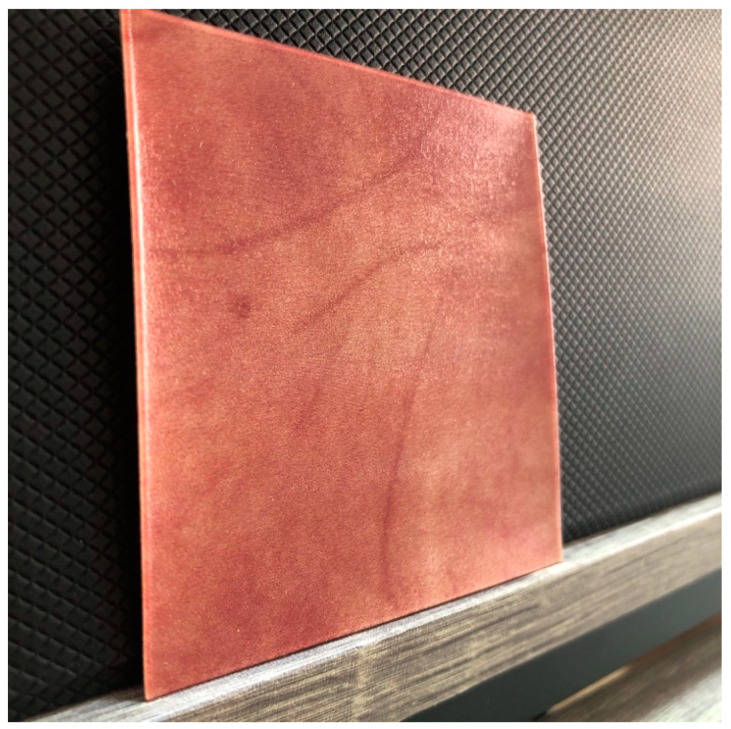
The fabrication result using multiple regression analysis.

The results using each method are shown in Figure 9. In this experiment, only one image of a palm was used, and it was of a man’s right hand. The target skin image is shown in Figure 9a, and Figure 9b–d shows the skin objects based on the CMYK concentrations estimated using LUT, multiple regression analysis, and neural networks, respectively. Subjectively, the method using multiple regression analysis yielded the most favorable results. The objects using LUTs lost their overall smoothness, which may be because of the lack of interpolation between the pigment concentrations obtained from the color patches. In the LUT, a data point outside the dataset was substituted by the closest value in the dataset. In the neural network-based method, the overall concentration was averaged and contrast was reduced. We consider that the neural network with three intermediate layers was overcomplicated because the present dataset was very linear. At least one layer of neural networks works similarly to multiple regression analysis, and thus the results would be comparable. We also evaluated the estimation accuracy of each of the three methods using leave-one-out cross-validation, and found that the RMSE of multiple regression analysis was minimal at 0.031. Significant differences in RMSE values for each method were analyzed by *t*-test. The results of the analysis at a significance level of 0.05 showed a significant difference in errors between multiple regression analysis and neural networks, whereas there was no significant difference in errors between multiple regression analysis and the LUT, and between neural networks and the LUT. Because of the high reproducibility of the detailed textures and the low error of the prediction method, we decided to use multiple regression analysis in this study. Here, white streaks are seen on the printed objects; however, since they are not observed in the halftone data for printing, we consider this as a problem with our printer. Figure 10 shows the fabricated object with multiple regression analysis. In addition, since our research aims at planer printing, we used a UV printer (UJF-3042HG, Mimaki Engineering, Nagano, Japan); however, the process is the same as a 3D printer. While Mimaki 3D printers [34] and Stratasys 3D printers [35], which are widely used for color 3D printing, have a resolution of 600 dpi, Mimaki’s UV printer can print at 1200 dpi vertically and 720 dpi horizontally. The higher the printing resolution of the printer compared to the resolution of the camera, the more halftone dots can be used to represent each pixel. Therefore, the higher printing resolution allows more detailed representation of the pigment concentration. Finally, we discuss the dependency of our method on printers and inks. The elements required for the method are full-color printing with CMYK inks, UV-curable inks that can be stacked, and clear inks. Therefore, we used a Mimaki UV printer (UJF-3042HG, Mimaki Engineering) as a printer setup that meets these requirements. There are several other UV printers that also satisfy the requirements (e.g., SC-V7000, Epson; VersaUV LEF2-200, Roland). In addition, the properties of clear ink vary depending on the ink manufacturer and may affect our method; thus, it is necessary to investigate the feasibility of using each clear ink through experiments.

### 6.2. Number of Clear Layers

As mentioned earlier, in addition to the melanin and hemoglobin layers, which are coloring layers, clear layers were inserted in the following order: the top layer, between the melanin and hemoglobin layers, and between the hemoglobin and reflective layers. We refer to these clear layers as the first, second, and third clear layers from the top, respectively. The first clear layer was used to protect the colored layer. The second and third clear layers were provided under the assumption that they improve skin-like appearance. We varied the number of layers (and thus the thickness) of the second and third clear layers to observe how they affect the skin-like appearance. We consider that the appearance is affected by the change in the thickness of the clear layer, which changes the depth of the pigment. Second and third clear layers with two, four, or six constituent layers were prepared, and nine samples were created with different combinations of these thicknesses, as shown in Table 1. The scattering characteristics of Mimaki’s clear inks have been investigated in the references [25], and it has been shown that a small amount of scattering occurs. Therefore, changing the thickness of the clear layer may cause inconsistency with the data of 900 color patches; however, in this study, we ignored this effect as it is very small. In this study, the layers of melanin and hemoglobin were fabricated in a single layer because the concentration was expressed in area gradation. In the future, we believe that it will be possible to represent subsurface scattering by spreading the pigments in the depth direction as well.

## 7. Subjective Evaluation

### 7.1. Evaluation Method

In this section, we evaluate the usefulness of our method in comparison with conventional methods. For subjective evaluation, we used the semantic differential method (SD method). This is a method proposed by the American psychologist Osgood to measure the impression of a target concept. The SD method uses pairs of adjectives that have opposite meanings (e.g., “rough” and “smooth” in our case). The evaluation was conducted using a slide bar with a scale of −5 to +5.

The 13 pairs of adjectives used in this experiment are shown in Table 2. The subjects were nine men and women in their twenties. The nine models shown in Table 1 were evaluated. In addition, to show the effectiveness of our method, we compared models with one and with two colored layers (all clear layers are a single layer). A typical one layer sample was printed by converting the RGB image of the target palm into CMYK values using Japan Color 2011 Coated, a color profile for printing, and inputting it into a same printer, Mimaki UJF-3042HG. In the future, we consider that it is necessary to compare with other commercial printer drivers. Experiments were conducted under daylight illumination using a lighting booth (Spectralight 2, Macbeth). The factors that contribute to the evaluation of skin appearance were extracted through factor analysis of the experimental data as the first factor. Factor analysis is a method used to find potential common factors from multivariate data, but the number of factors first needs to be determined. Therefore, we evaluated the factor loadings, which indicate the contribution rate of each factor, and found that up to the third factor, the contribution rate exceeded 90%. Based on this result, the number of factors was set to 3.

### 7.2. Evaluation Results

Based on the factor analysis, the first factor was defined as the skin-ness factor. Evaluation of this factor was obtained by taking the weighted average of the evaluation values using the factor loadings. For a conventional one-layer model and our two-layer model, this factor was −2.37 and 0.06, respectively. Thus, it can be seen that our proposed method is able to more realistically reproduce skin appearance. The results of the evaluation of the thickness of the clear layers (defined in Table 1) are presented in Table 3. The highest values were obtained when the second clear layer comprised two layers and the third clear layer comprised four layers. The evaluation value tended to increase as the number of the third clear layer increases. However, since there are exceptions, we consider that it is necessary to conduct experiments with more subjects to reduce the effect of the individual differences and obtain more accurate results.

## 8. Conclusions

In this paper, we proposed a pipeline for modeling of human skin with multilayered spatial distributions of melanin and hemoglobin components based on images taken by an RGB camera. To obtain pigment concentration distributions from the RGB skin images, pigment component separation was used. Each pigment concentration was then converted to a CMYK value using a color patch-based method. Halftoning was applied, and a clear layer was inserted to realistically reproduce human skin. Subjective evaluation experiments showed that our method could reproduce a more skin-like texture than the conventional printing method. We also investigated the effect of varying the thickness of the inserted clear layer on the appearance. As a result, it was shown that changing the thickness of the clear layer affects the appearance, and the combination with the highest evaluation value was determined among the nine combinations of clear layer thicknesses prepared for this study.

A remarkable feature of our method is the use of pigment concentration for the reproduction of skin tones in printing. This method can be used to represent natural skin tone changes by varying the pigment concentration, or to observe the relationship between pigment and appearance on a real object. Here, a limitation of this study is that the objects that can be fabricated are limited to planar objects. Therefore, as future works, our method should be improved to be applied to 3D geometry and objects.

## Figures and Tables

**Table 1:** Number of layers of each clear layer in the different samples.

Sample No.	1	2	3	4	5	6	7	8	9
**2nd clear layer**	**2**	**2**	**2**	**4**	**4**	**4**	**6**	**6**	**6**
**3rd clear layer**	**2**	**4**	**6**	**2**	**4**	**6**	**2**	**4**	**6**

**Table 2:** Adjective pairs used in the evaluation.

Negative	Positive
**Rough**	**Smooth**
**Rough**	**Slippery**
**Not-slippery**	**Slippery**
**Dark**	**Bright**
**Sober**	**Brilliant**
**Mat**	**Glossy**
**Heavy**	**Light**
**Nonelastic**	**Elastic**
**Dry**	**Wet**
**Hard**	**Soft**
**Cold**	**Hot**
**Not transparent**	**Transparent**
**Artificial**	**Natural**

**Table 3:** Evaluation results for the thickness of clear layers.

Sample No.	Number of 2nd Clear Layers	Number of 3rd Clear Layers	Evaluation Value
**1**	**2**	**2**	**0.9428**
**2**	**2**	**4**	**1.5186**
**3**	**2**	**6**	**0.6254**
**4**	**4**	**2**	**−0.5796**
**5**	**4**	**4**	**0.9439**
**6**	**4**	**6**	**1.3705**
**7**	**6**	**2**	**−0.0477**
**8**	**6**	**4**	**1.3850**
**9**	**6**	**6**	**1.4106**
